# Measures of high-density lipoprotein function in men and women with severe aortic stenosis

**DOI:** 10.1186/s12944-022-01653-7

**Published:** 2022-05-28

**Authors:** Anouar Hafiane, Elda Favari, Anna E. Bortnick

**Affiliations:** 1 Department of Medicine, Faculty of Medicine, Research Institute of the McGill University Health Centre, 1001 Boulevard Decarie, Montreal, Québec H3A 1A1 Canada; 2 Department of Food and Drug, University of Parma, Parma, Italy; 3 Department of Medicine, Division of Cardiology, Bronx, New York, USA; 4 Division of Geriatrics, Montefiore Medical Center and Albert Einstein College of Medicine, Bronx, New York, USA

**Keywords:** High-density lipoprotein, Cholesterol efflux capacity, Valve, Aortic stenosis, Calcification

## Abstract

**Background:**

Calcification of the aortic valve is a common heart valve disorder, in some cases leading to clinically impactful severe aortic stenosis (AS). Sex-specific differences in aortic valve calcification (ACV) exist, with women having a lower burden of calcification than men as measured by computed tomography; however, the pathophysiological mechanism that leads to these differences remains unclear.

**Methods:**

Using cultured human Tamm-Horsfall protein 1 (THP-1) macrophages and human aortic valve interstitial cells, the effects of high-density lipoprotein (HDL) particles isolated from the plasma of men and women with severe AS were studied for cholesterol efflux capacity (CEC).

**Results:**

HDL-CEC was assessed in 46 patients with severe AS, *n* = 30 men, *n* = 16 women. ATP-Binding Cassette A1 (ABCA1)-mediated HDL-CEC was measured from human cultured THP-1 macrophages to plasma HDL samples. Women with severe AS had more ABCA1-mediated HDL-CEC, as compared to men (8.50 ± 3.90% cpm vs. 6.80 ± 1.50% cpm, *P* = 0.04). HDL pre-β1 and α-particles were higher in woman than in men by spectral density, (pre-β1 HDL, 20298.29 ± 1076.15 vs. 15,661.74 ± 789.00, *P* = 0.002, and α-HDL, 63006.35 ± 756.81 vs. 50,447.00 ± 546.52, *P* = 0.03). Lecithin-cholesterol acyltransferase conversion of free cholesterol into cholesteryl esters was higher in women than men (16.44 ± 9.11%/h vs. 12.00 ± 8.07%/h, *P* = 0.03).

**Conclusions:**

Sex-specific changes in various parameters of HDL-CEC were found in patients with severe AS. Sex-based modifications in HDL functionality by HDL-CEC might account for the reduced burden of calcification in women vs. men with severe AS. Therefore, future studies should target sex-related pathways in AS to help to improve understanding and treatment of AS.

**Graphical abstract:**

Sex specifc differences in AVC and differences associated with HDL function in men and women with severe AS. When compared to men, women had higher preβ-HDL and α-HDL migrating particles, higher cholesterol efflux to HDL, and higher lecithin cholesterol acyl transferase (LCAT) activity, possibly indicating that improved reverse cholesterol transport may be protective against worsened calcification. 
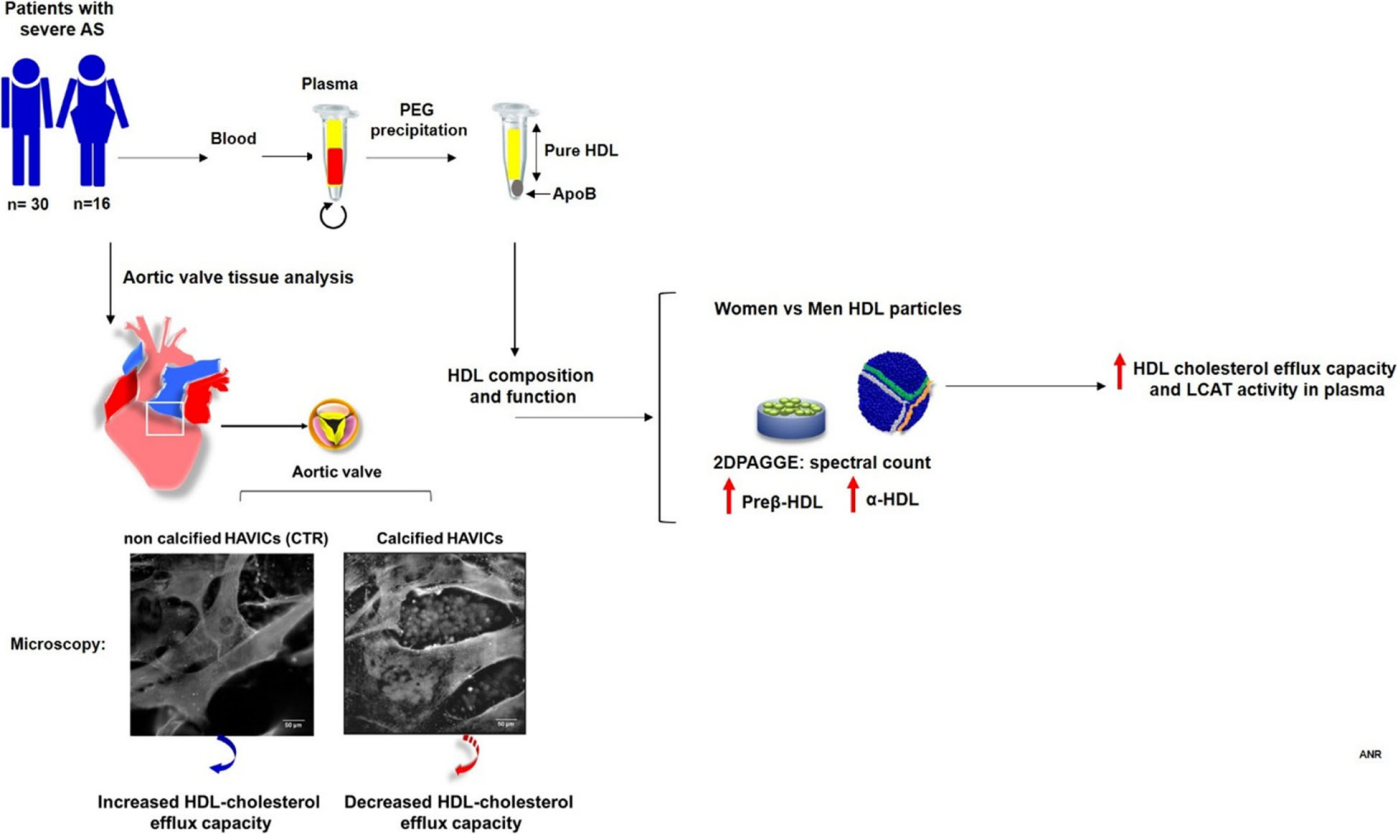

**Supplementary Information:**

The online version contains supplementary material available at 10.1186/s12944-022-01653-7.

## Introduction

Aortic stenosis (AS) disease is one of the most common valvular diseases associated with age [1, 2]. Over the past 30 years, the global prevalence of calcific aortic valve disease-associated deaths has increased [3]. There is no medical therapy to slow the progression of AS, at present. Patients with severe AS are treated with either surgical valve or transcatheter prosthesis [4]. Aortic valve calcification (AVC) shares several pathophysiologic resemblances with atherosclerosis, including the migration of inflammatory cells into tissue and the development of calcium deposits [5–7]. Clinical and Mendelian randomization studies affirm similarities between AVC and atherosclerosis, suggesting overlapping causal risk factors [8, 9]. Male sex, elevated serum low-density lipoprotein (LDL)-cholesterol, age, elevated lipoprotein (Lp) (a), hypertension, diabetes, renal failure, and smoking are all associated with AS [9–11]. Several randomized clinical trials of lipid-lowering therapy with statins indicated no effect on valve structure, function, or calcification. This is potentially because statins increase Lp (a), and effective medical therapy to prevent the progression of AVC remains elusive [8]. Women with severe AS are known to have a lesser burden of calcification than men as measured by computed tomography and lower valve weight on examination after surgical explant [4, 12–15]. Some potential sex-specific mechanisms which might affect calcification involve the pro-calcific effect of testosterone, altered inflammatory responses, as well as differential expression of calcification inhibitor proteins [16–18]. Limited evidence suggests no link between HDL function and AS [19]. Since AS is more prevalent in men than in women, evaluation of AS by sex may help to understand differences in the burden of the disease. ABCA1-mediated cholesterol efflux capacity (CEC), as a measurement of high-density lipoprotein (HDL) functionality, is associated with protection against cardiovascular events [20–23]. ABCA1 is a membrane transporter that promotes cellular cholesterol efflux by transfer of free cholesterol and phospholipid to apolipoprotein (apo) A-I, an early step in nascent HDL formation which is atheroprotective [24]. Whether variation in HDL-CEC, HDL particles size (nm), or protein composition slow the development of AS is unknown. One animal study demonstrated regression in AS severity with apo A-I mimetic peptide therapy [25]. Other studies have shown that HDL might play a role in AS, and HDL-associated proteins have been detected in AS tissue [19, 26–28]. The relationship of HDL-CEC to AS is under-studied. The present study hypothesized that HDL-CEC might be 1) dysregulated from calcified human aortic valve interstitial cells (HAVICs), (the main cell type involved in the progression of AVC), and 2) a protective mechanism that is upregulated in women with AS vs. men.

## Material and methods

### Human aortic valve interstitial cells, isolation, and culture

Plasma samples were obtained from patients with severe AS (*n* = 46) on the day of valve replacement. Blood specimens were obtained from the study subjects by venipuncture; plasma was separated (2000 g, 4 °C for 20 minutes) and placed in − 80 °C in 0.5 mL aliquots until use. Control HDL was collected from 6 healthy men and women, pooled, and stored at − 80 °C until use in optimizing the HDL-CEC assay kinetics from Tamm-Horsfall protein 1 (THP-1) macrophages and HAVICs. In this study, human primary calcified and non-calcified cells were obtained from explanted aortic valves of men with severe AS, as previously reported [29]. Briefly, fresh aortic valve (AV) leaflets were washed with 1x Hanks’ Balanced Salt Solution (HBSS) buffer (Thermo Fisher Scientific, Waltham, Mass), and manually debrided to remove endothelial cells. The digested tissue mixture was centrifuged at 500 g at 4 °C for 10 min, and the supernatant was transferred into a new tube and centrifuged at 1000 g at 4 °C for 10 min. Cell pellets were suspended in DMEM high glucose, 10% fetal bovine serum (FBS), and seeded in culture flasks until confluence (which took 9 to 14 days). HAVICs in passages 2 to 5 were used for all experiments. Cells were trypsinized and plated for each experiment at a density of 6.0 × 10^4^ cells in a 24-well plate. Participants provided informed consent. The study was approved by the McGill University Institutional Review Board (IRB). The lipid characteristics, the ejection fraction (%), the maximum pressure across the valve (Pmax), and the mean pressure across the valve (Pmean) are noted in Table 1.

**Table 1:** Baseline clinical characteristics of men and women with severe AS (*n* = 46)

Clinical parameter	Men (***n*** = 30)	Women (***n*** = 16)
Age, (years)	70 (10)	68 (9.5)
BMI, kg/m^2^	25.19 (5.09)**	28.3 (8.25)*
Total Cholesterol, mmol/L (mg/dL)	4.8 (0.93) 189.6 (36)	5.3 (2.76) 205.7 (106.70)
TG, mmol/L (mg/dL)	1.5 (1.44) 132.0 (127.60)	2.2 (1.14)* 198.4 (101)
HDL-C, mmol/L (mg/dL)	1.2 (0.60) 47.2 (23.20)	1.3 (0.51) 48.8 (19.72)
LDL-C, mmol/L (mg/dL)	2.6 (0.70) 101.7 (27.23)	3.5 (2.49)* 136.7 (96.30)
Lp(a), μg/mL	102.0 (53)	118.4 (44)
Left ventricular ejection fraction, (%)	57.0 ± 12.86	54.0 ± 14.52
Pmax, mm Hg	58.2 ± 28.56**	50.5 ± 18.3
Pmean, mm Hg	32.8 ± 16.46*	28.5 ± 10
α-HDL size, (nm)	5.9 ± 0.60	6.7 ± 2.38
Preβ1-HDL, total spectral count	15,661.7 ± 789	20,298.3 ± 1076.15*
α-HDL, total spectral count	50,447.0 ± 546.52	63,006.0 ± 756.80**
Cholesterol Efflux, %	7.1 ± 0.38	8.2 ± 0.47**
LCAT activity, %	12.0 ± 8.07	16.5 ± 9.11*

Continuous variables are presented as mean ± standard deviation or as median (interquartile range) if skewed. For cholesterol conversion to mg/dL multiply by 38.67 and for triglycerides, multiply by 88.57

*BMI* body mass index, *HDL-C* high-density lipoprotein cholesterol, *Pmax* maximum pressure across the valve, *Pmean* mean pressure across the valve, *LCAT* Lecithin–cholesterol acyltransferase, *LDL-C* low-density lipoprotein cholesterol, *Lp(a)* lipoprotein(a), *TG* triglycerides

*P < 0.05, **P < 0.01

### Immunofluorescence

To demonstrate ABCA1 upregulation in HAVICs within the HDL-CEC process, calcified and non-calcified HAVICs were treated with 2 μCi/mL^3^[H]-cholesterol in presence of 1% FBS for 48 h at 37 °C and incubated with purified HDL from healthy individuals for 24 h. Cells were then washed with cold PBS, and fixed with 4% paraformaldehyde at room temperature for 15 minutes. Cells were incubated with anti-α-smooth muscle actin antibody (1:200; Abcam, Ontario, Canada) and anti-ABCA1 antibody (1:200, Novus Biological, Ontario, Canada). Afterward, a fluorophore-conjugated secondary antibody (1:250; Thermo Fisher Scientific, Waltham, Mass), and NucBlue (Thermo Fisher Scientific, Waltham, Mass), were added respectively. Cell images were made by Olympus microphotographic system (Carl Zeiss Canada Ltd., Ontario, Canada) confocal microscope.

### Western blotting analysis

Lysis buffer (20 mM Tris, 5 mM EDTA, and 5 mM EGTA; pH 7.5) containing protease inhibitor mixture (one tablet/50 mL, Roche) was used to lysis HAVICs. The suspension was subjected to further lysis in the presence of cOmplete™ EDTA-free Protease Inhibitor Cocktail (Sigma Aldrich, Ontario, Canada) followed by 1500 g centrifugation at 4 °C for 10 min. Samples were immunoblotted using affinity-purified human anti-ABCA1 (Novus Biological, Ontario, Canada). Membrane pore size 0.45 μm (Amersham, Darmstadt, Germany) was re-probed with Glyceraldehyde 3-phosphate dehydrogenase (GAPDH) (Abcam, Ontario, Canada) as a loading control. Molecular weight protein standard mixture (10–250 kDa) from (Bio-Rad, Hercules, California, US) was included. Bands were revealed by chemiluminescence reagent (ZmTech Scientifique, QC, Canada).

### Cholesterol efflux capacity assays

HDL-CEC assays were performed using apolipoprotein B-depleted plasma (plasma HDL, 2.8%) as previously reported [22, 23, 30]. HDL was purified by polyethylene glycol (PEG, MW8000, Sigma, Oakville, Canada) 20% (40:100 plasma, vol/vol) and diluted to 2.8% in minimal essential medium-HEPES (0.5 mL/well). THP-1 human monocyte cell line (American Type Tissue Culture Collection, Camden, NJ), were plated in 24 well-plates in Roswell Park Memorial Institute (RPMI) 1640 medium containing 10% FBS, 50 μg/mL gentamicin, and maintained at 37 °C in a humidified atmosphere of 5% CO_2_ [31]. Differentiation of THP-1 monocytes into macrophages was induced via addition of 200 nM phorbol 12-myristate 13-acetate (PMA, Sigma-Aldrich, Ontario, Canada) for 72 h before experiments [31]. THP-1 human macrophages were incubated with 2 μCi ^3^[H]-cholesterol (Perkin Elmer, Norwalk, Connecticut) for 24 h, 1% FBS. ABCA1 protein expression in THP-1 macrophages was upregulated with 10 μM 9-cis-retinoic acid (9cRA) plus 5 μg/mL 22-hydroxycholesterol (22-OH) for 16 h, in RPMI-1640 medium containing 0.2% bovine serum albumin (BSA). HAVICs were cultured in Dulbecco’s Modified Eagle’s Medium (DMEM), 10% FBS, 100 U/mL penicillin, and 100 U/mL streptomycin (Penicillin-Streptomycin, Invitrogen, Ontario, Canada) and 5% glucose. Medium and cell-associated ^3^[H]-cholesterol were subjected to liquid scintillation counting. HDL-CEC was determined as % cpm of ^3^[H]-cpm in media / (^3^[H]-cpm in media + ^3^[H]-cpm in cells). Each patient sample was run in triplicate. The coefficient of inter variability was 4.39%. Cholesterol efflux efficiency (*K*_*m*_) and maximum velocity (*V*_*max*_) were calculated by fitting plots of the fractional 24 h lipid efflux against purified plasma HDL concentrations with 0.5 to 24% plasma HDL/mL.

### Characterization of nascent HDL particles

Two-dimensional non denaturing gradient gel electrophoresis (2D-PAGGE) was performed to characterize apoA-I-containing particles from HDL isolated from men (*n* = 10) and women (*n* = 10) as previously reported [32]. The conditioned medium from HAVICs exposed to HDL was concentrated by a size-exclusion centrifugal filter (molecular weight cutoff (MWCO) 30,000; Amicon, Merck Millipore Co., Cork, Ireland) that separates between lipid-free apoA-I and other lipidated (LpA-I) particles. ApoA-I proteins were probed with an anti-apoA-I antibody (Biodesign, Meridian Life Science, Tennessee, USA). A standard native high-molecular-weight (4–17.0 nm) from (GE Healthcare, UK) was used in each gel and revealed by Ponceau S sodium salt.

### Lecithin-cholesterol acyltransferase (LCAT) activity assay

**Fig. 1:**
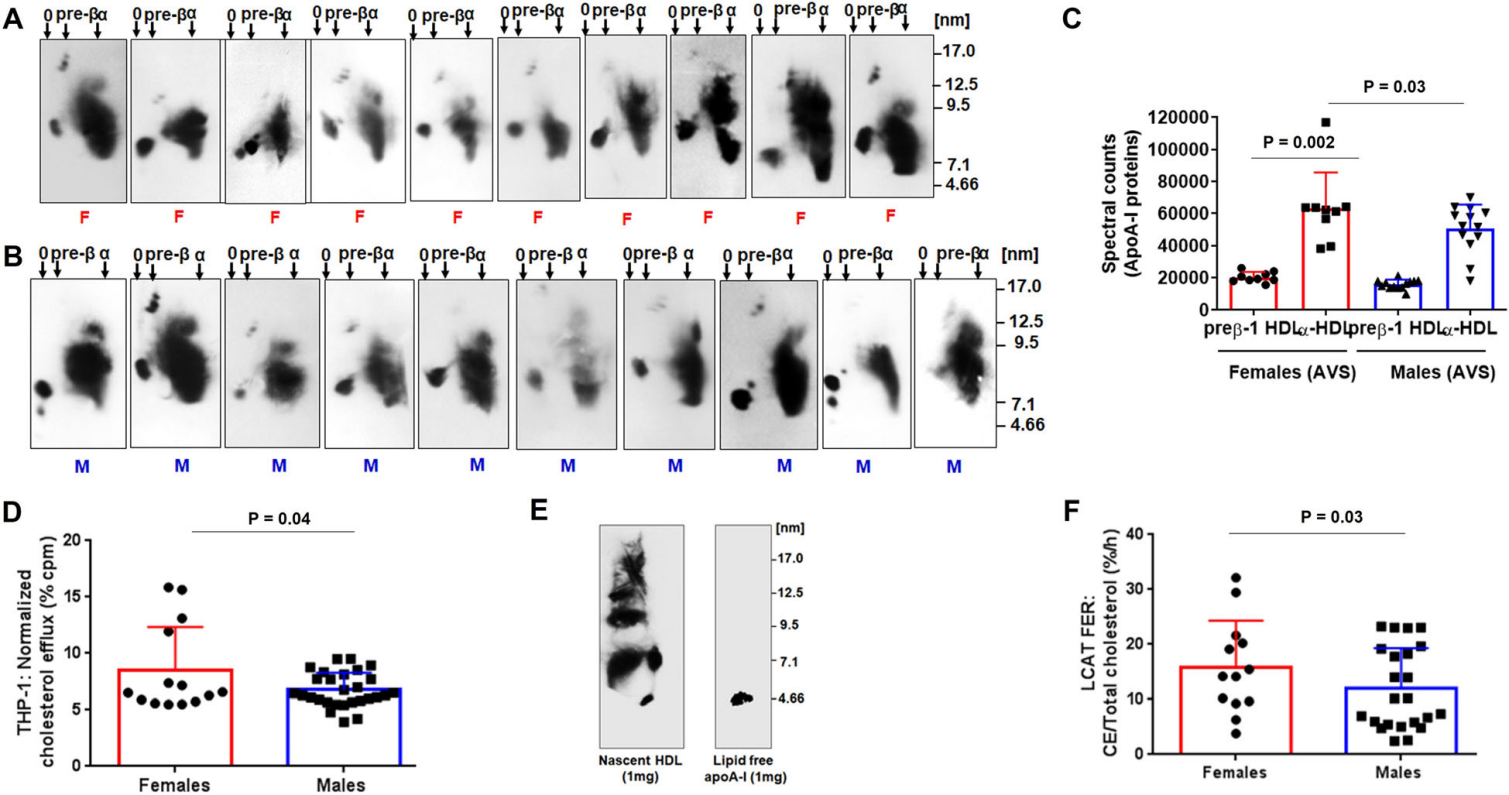
Measurement of high-density lipoprotein (HDL) function in patients with severe AS. HDL particles were isolated by PEG from women (F, red, **A**) and men (M, blue, **B**) with severe AS. ApoA-I containing particles were probed with human anti-apoA-I antibody and revealed by chemiluminescence. Molecular size markers are shown. (**C**) ApoA-I preβ1-HDL and α-HDL particles were quantified by Western blotting followed by densitometric scanning. **(D)** ABCA1-mediated cholesterol efflux capacity to HDL from women (red) and men (blue) with severe AS. (**E**) Formation of lipidated apoA-I particles from BHK cells expressing ABCA1 and separation by 2D-PAGGE. (**F**) LCAT activity (%/h) in plasma samples. After lipid extraction, ^3^[H]-unesterified cholesterol and ^3^[H]-cholesteryl ester were separated by thin liquid chromatography and assayed for radioactivity

A proteoliposome substrate of apoA-I: ^3^[H]-cholesterol nascent HDL(nHDL)-apo A-I was prepared to determine LCAT activity in plasma samples, as previously described [33]. nHDL-apo A-I particles were prepared after radiolabeling of baby hamster kidney (BHK) cells with ^3^[H]-cholesterol for 24 h in DMEM 10% FBS. Afterward, cells were incubated with DMEM/BSA for 6 h and then incubated with 15 mL of DMEM/BSA containing 10 nM mifepristone (Invitrogen, Carlsbad, CA, USA) and 10 μg/mL apo A-I for 18 h. The medium was centrifuged at 1500 g at 4 °C for 10 min to remove cell debris. Lipid free apoA-I was removed by a 50 kDa column and dialyzed extensively against phosphate-buffered saline (PBS). The substrate (nHDL-apo A-I) was used to determine LCAT activity as the fractional esterification rate in plasma from AS patients. The lipidated HDL particles were verified by qualitative 2D-PAGGE before and after incubation in total plasma (Fig. 1A). LCAT activity in nHDL-apoA-I was used as positive control and LCAT inhibitor 5,5′-dithiobis (2-nitrobenzoic acid) (DTNB), 5 mM from (Sigma-Aldrich, Ontario, Canada) was used as a negative control [34]. Cellular lipids were extracted and ^3^[H]-cholesterol and ^3^[H]-cholesteryl esters were separated by thin-layer chromatography (TLC). Lipid species (free cholesterol and cholesteryl ester) were visualized with iodine vapor (Fisher Scientific, Ontario, Canada), and lipids spots were scraped into liquid scintillating vials and were assayed for radioactivity. The LCAT activity was calculated as the fractional esterification rate of cholesterol (%/h) in plasma, expressed as the ratio of radioactive unesterified to radioactive esterified cholesterol on nHDL [35].

### Statistical analyses

Variables are presented as mean ± standard deviation for normally distributed variables or median and interquartile range for non-normally distributed variables. A non-parametric test (Mann-Whitney) was used to compare skewed distributions and the unpaired Student’s *t*-test was used to compare the differences between normally distributed groups. The data were carried out by using GraphPad Prism version 6 (La Jolla, California). *P* < 0.05 (2-tailed) is statistically significant.

## Results

### HDL functionality is reduced in men as compared to women with severe AS

Women with severe AS were similar in age to men with comparable Lp (a) levels, but were more likely to be overweight, and had higher triglycerides and LDL cholesterol (Table 1). Quantification of apoA-I-containing HDL subpopulations by spectral density (Fig. 1A, B) showed that women had more abundant pre-β1- and α-HDL than men (Fig. 1C) by spectral density and size. Women with severe AS had statistically significant higher ABCA1-mediated efflux to plasma HDL from stimulated human THP-1 macrophages than men (Fig. 1D). LCAT activity using an artificial proteoliposome substrate of (apo) A-I:^3^[H]-cholesterol:lecithin (Fig. 1E), was higher in women with severe AS compared to men (Fig. 1F). Obesity has been associated with differences in HDL-CEC [36], but in this small sample, CE was poorly correlated with body mass index (BMI) (*r* = 0.36, *P* = 0.86).

### Calcification process reduced ABCA1 expression in HAVICs

**Fig. 2:**
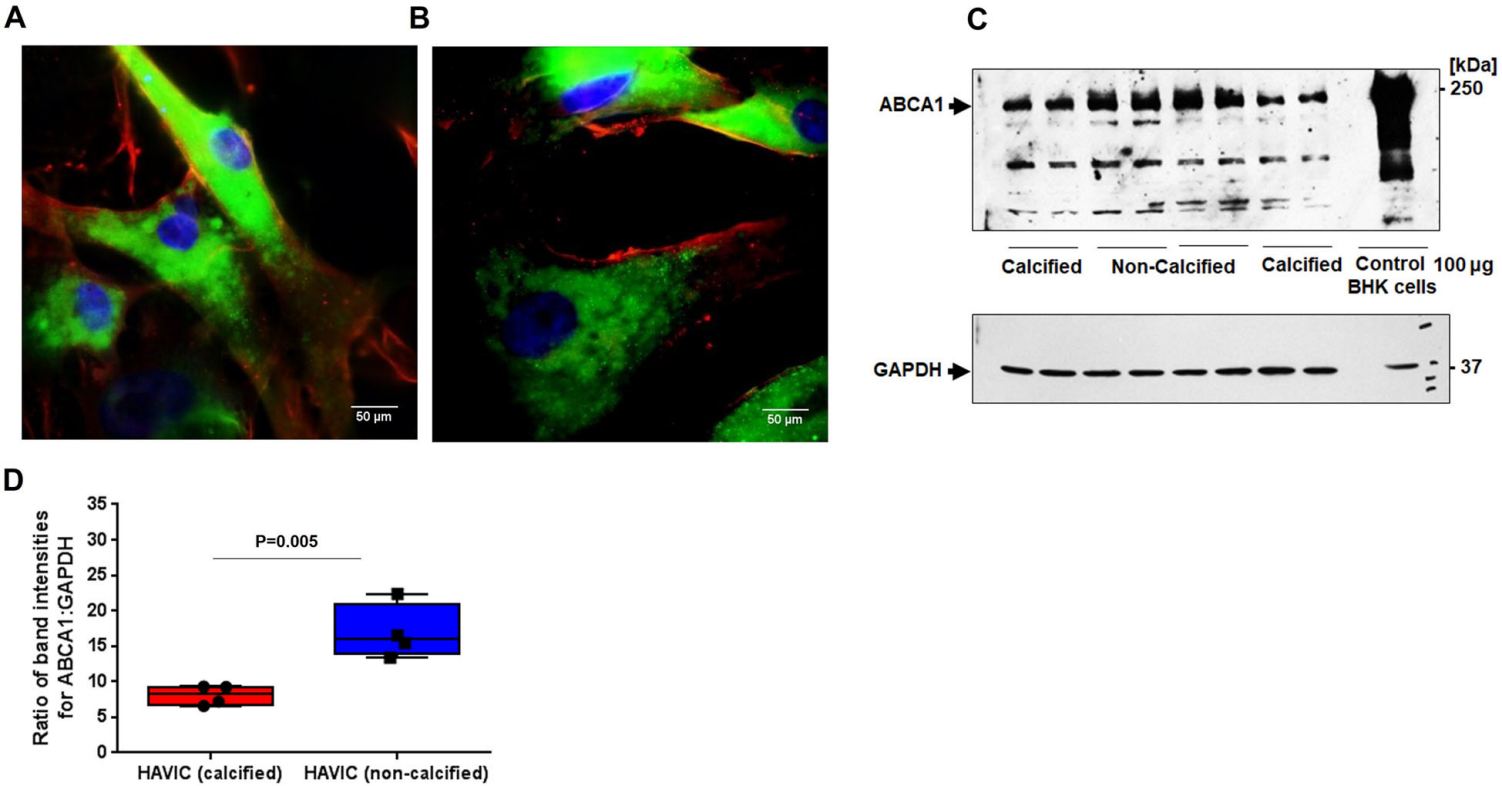
Characterization of human aortic vascular interstitial cells (HAVICs). (**A**, **B**) Marker for HAVICs in culture, alpha-smooth muscle actin (α-SMA), and ATP-Binding Cassette A1 (ABCA1) protein by immunofluorescence. Immunofluorescence staining with α-SMA (green) and ABCA1 (red) in (**A**) calcified and (**B**) non-calcified HAVICS are shown. Nuclei were stained (blue) with 4′,6-diamidino-2-phenylindole (DAPI). (**C**) ABCA1 protein expression in BHK cells was used as a control. Cells (100 μg) were separated by SDS-PAGE (8–28%) and probed for ABCA1 detection. (**D**) Data are expressed as the ratio of quantified band intensities for ABCA1 and GAPDH proteins

Extraction of primary HAVICs from calcified and non-calcified regions was performed from aortic valve leaflets of the same patient. The phenotype of primary HAVICs was confirmed with immunofluorescence by probing α-smooth muscle actin (α-SMA, green), as previously described [37]. Cultured HAVICs expressed α-SMA (green) and ABCA1 (red) in non-calcified (Fig. 2A) and calcified cells (Fig. 2B). The western blotting analysis demonstrated that calcified HAVICs expressed less ABCA1 protein than non-calcified cells (Fig. 2C). ABCA1 amounts were significantly decreased by 49 ± 14% in calcified vs. non-calcified HAVICs (*P* = 0.01), as confirmed by spectral analysis (Fig. 2D).

### Calcification of HAVICs reduces cholesterol efflux to control plasma HDL

**Fig. 3:**
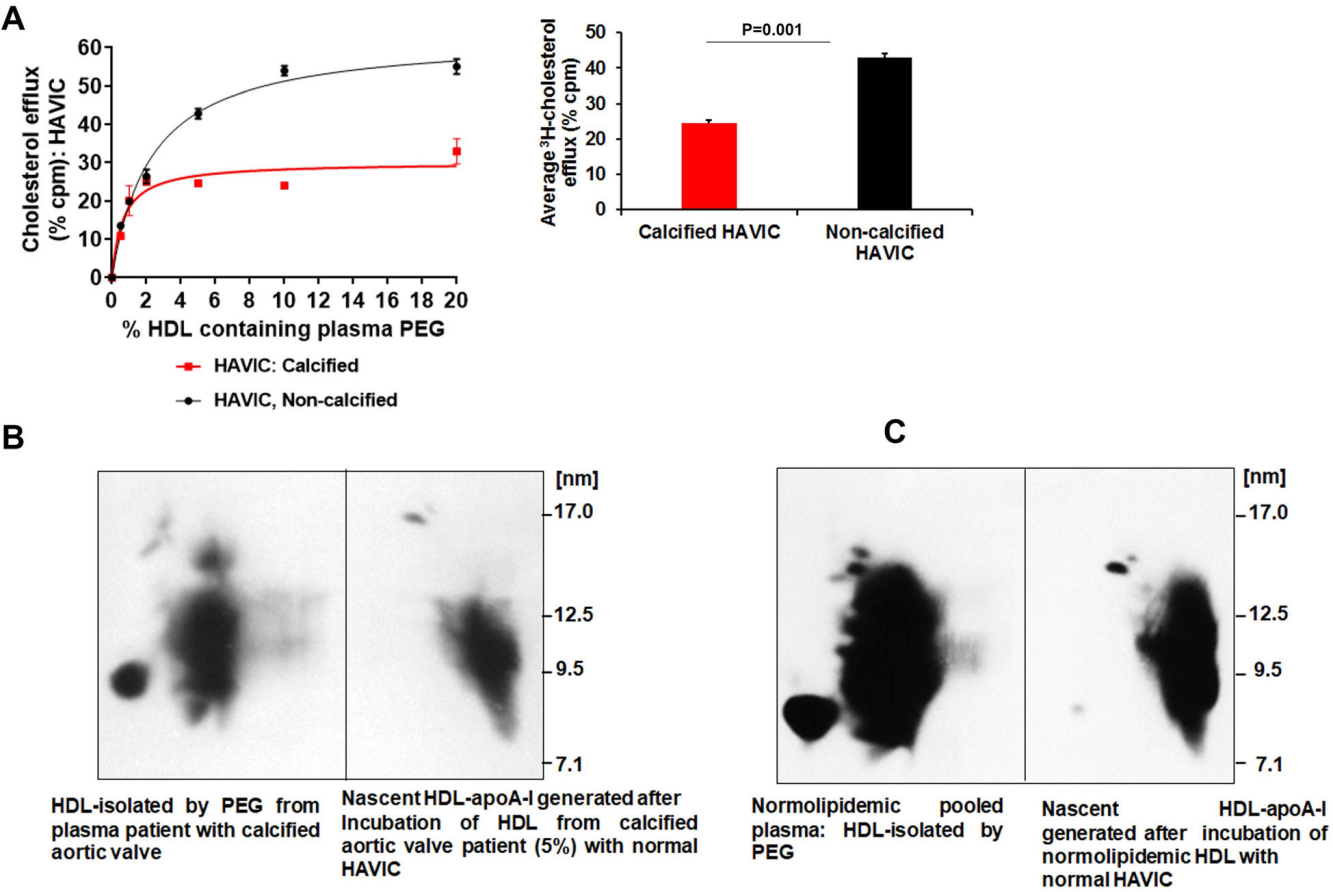
Cholesterol efflux to control HDL is reduced in calcified human aortic vascular interstitial cells (HAVICs) in a dose dependent manner. (**A**) After incubation, with ^3^[H]-cholesterol 2 μCi/mL for 24 h in DMEM 1% FBS HAVICs (calcified and non-calcified) were washed twice with PBS. Cells were incubated with increased doses of pooled control, high-density lipoprotein (HDL) from healthy individuals for 24 h. (*Inset*) Cellular cholesterol efflux at 5% plasma HDL. Characterization of cholesterol efflux from calcified HAVICs. Radiolabelled HAVICs, Calcified (**B**) vs. non-calcified (**C**), were incubated with 5% plasma control HDL for 8 h. Media from cells samples were collected, concentrated and separated by 2D-PAGGE, and apoA-I was detected by Western blotting. Molecular size markers are shown. HDL, high-density lipoprotein; HAVIC, human aortic interstitial cells

The HAVICs were used as an in vitro model to understand the effect of calcification on CE, as this cell type is central to the progression of AVC [27]. CEC from HAVICs and THP-1 macrophages was optimized to determine the optimal concentration and timing of incubation with purified control plasma HDL (Supplemental Material, Fig. 1A-D). An HDL concentration of 5% (Supplemental Material, Fig. 1A) and 2.8% (Supplemental Material, Fig. 1C) and 8 h (Supplemental Material, Fig. 1B, D) were considered optimal for HAVICs and THP-1 macrophages, respectively. The ability of calcified HAVICs to perform cholesterol efflux and generate nascent HDL particles from incubation with plasma HDL were also characterized. HDL-CEC values were compared from calcified HAVICs and non-calcified HAVICs incubated with control plasma HDL and analyzed nascent particles by 2D-PAGGE (Fig. 3A, B, and C). Cholesterol efflux from calcified HAVICs to increasing doses of control plasma HDL was decreased as compared to non-calcified cells (Fig. 3A, *inset*). This was associated with reduced α-nascent HDL particles when compared to non-calcified HAVICs (Fig. 3B, C right panel). During cholesterol efflux from THP-1 and HAVICs, calcified HAVICs performed cholesterol efflux with lower *K*_m_ efficiency and less accelerated *V*_max_ vs. non-calcified cells or THP-1 macrophages (Supplemental Material, Table 1).

## Discussion

In this study, ABCA1 expression and cholesterol efflux from calcified HAVICs, the main cell type implicated in AVC, as well as sex-specific differences in HDL particle size, HDL-CEC, and LCAT activity from men and women with severe AS were evaluated. Of relevance, expression of ABCA1 in calcified HAVICs was reduced as compared to non-calcified cells and they had slower, and less efficient cholesterol efflux to control plasma HDL. In the present study women with severe AS had more pre-β1 nascent HDL particles, which are known to interact well with ABCA1 to modulate cholesterol efflux [38]. Herein, women with severe AS had higher HDL-CEC and LCAT activity. Evidence indicates a delayed rate and decreased absolute amount of valvular calcification in women as compared to men with severe AS, but the reasons for this are not well elucidated [4, 13, 14]. AS shares common risk factors and has similar pathophysiology to coronary artery disease. Decreased cholesterol efflux from calcified HAVICs has been documented, but whether this occurs through ABCA1, other transporters, or alternative signaling pathways is unclear [39]. For instance, interferon-γ *receptor 1* expression and ERK/HIF-1α signaling were greater in HAVICs from men, a possible mechanism for amplified calcification, while matrix Gla protein, an inhibitor of calcification, and delayed osteogenesis were detected in aortic valve tissue from women [16, 17]. Calcified HAVICs have lower cholesterol efflux and accordingly lower ABCA1 protein expression than non-calcified HAVICs. Therefore, decreased expression of ABCA1 by calcified HAVICs may lead to reduced efficiency and rate of CEC (Graphical Abstract). Higher ABCA1-mediated efflux was previously linked to decreased AVC through administration of liver X receptor agonist T1317 [40]. Boosting ABCA1 expression reduced calcium and lipid deposition in the AV from hypercholesterolemic mice in vivo [40]. Sex-specific differences in LCAT activity have been previously documented in individuals with cardiovascular disease, and observations in the present study are now extend this to severe AS [41]. Increased LCAT activity is beneficial in reverse cholesterol transport because it remodels nascent HDL to more mature particles, which return cholesterol to the liver for excretion [42]. Specifically, α-HDL subspecies (12 to 17 nm), considered to be atheroprotective, are generally increased in women and are associated with higher LCAT activity [43]. Differences in HDL functionality between men and women might explain, in part, why women have less calcification in severe AS, but HDL may have other beneficial effects. To this end, HDL has been reported as having a direct anti-calcifying impact on human AV [26]. HDL stimulates osteoprotegerin in AV, a decoy receptor for receptor activator of nuclear factor-kappa B ligand (RANKL), which inhibits differentiation of cells into osteoclasts. HDL also reduces tumor necrosis factor-α, a driver of calcification, in AV [26]. HDL particles also carry a vast proteome which may account for the spectrum of anti-inflammatory and antioxidant properties as cholesterol acceptors, and future studies should focus on these properties in relation to protection against AV calcification [44]. Estrogen may protect against the development of valvular calcification, which could account for delayed accumulation in women as compared to men. Testosterone influenced the onset of AVC in an LDLr^−/−^/Apo B^100/100^/IGF-II mouse model, and sex-based differences in calcification were decreased with castration [18]. In relation to ABCA1, serum 17β-estradiol doses does not appear to influence efflux [45, 46]. An estrogen effect would not account for changes in HDL functionality in the present study, as women were of postmenopausal age. A previous study investigating the role of HDL in AS showed no difference in HDL measures between AS cases vs. age and sex-matched controls but did not stratify by sex or compare men and women with severe AS [19]. A Finnish study evaluating HDL-CEC in a small sample of healthy men and women demonstrated no difference by sex or menopausal status [47]. However, the study did not measure ABCA1-mediated HDL-CEC, but rather, total efflux to HDL from unstimulated cells, and women were grouped as pre-or post-menopausal by age, and their status was possibly mis-classified [47]. This study adds evidence to existing knowledge suggesting sex-specific dysfunctionality of HDL in severe AS in men as compared to women [13]. This was also reported more recently in moderate-to-severe AS, though not stratified by sex [30]. Another in vitro study showed that rodent valvular interstitial cells (VICs) behaved differently in a hormone-free environment, with VICs derived from males calcifying more extensively than VICs derived from females [48]. Data from this study, in combination with emerging evidence, supports that there are sex-specific modifications in HDL measures which might explain biological differences in the severity of calcification between men and women.

## Study strengths and limitations

The relationship of HDL-CEC with calcific aortic valve disease has not been well studied. Higher HDL-CEC in women may be an explanation for the lower calcific load of an equivalent clinical severity of AS. The major limitation of the present study is its small study sample and lack of HAVIC cultured cells from women to compare with those of men. Also, non-calcified areas of AS tissue were used to compare with calcified areas, which might have similar inflammatory or oxidative processes. Non-calcified valve tissue can also be obtained from cadavers or heart transplants, but proteins may be degraded post-mortem and transplant candidates likely have different demographic and clinical characteristics than AS patients, thus, the best control for AS tissue and cell culture studies is unclear. Lastly, additional research is required to assess the causality of the observed relationship and these findings would be strengthened through validation in larger cohorts.

## Conclusion

This study shows that HDL-CEC is significantly impaired in men with severe AS when compared to women. In addition, HDL-CEC from HAVICs supports a possible link between calcification and impaired ability to promote ABCA1-mediated efflux and HDL biogenesis. These data support future sex-stratified studies of the role of efflux in protection against AS incidence and progression. These findings need to be further linked to mechanisms of delayed calcification in women with severe AS and require validation in a larger cohort, perhaps with sub-stratification by prevalent co-morbidities to understand if there are additional interactions by age, and presence of atherosclerosis, or by varying degrees of calcification. In conclusion, sex differences in ABCA1-mediated HDL-CEC should be evaluated in larger studies of AVC progression and severe AS.

## Supplementary Information

**Additional file 1.**

## Data Availability

Data and materials are available on request.

## Abbreviations

AS: Aortic stenosis
AVC: Aortic valve calcification
ABCA1: Adenosine triphosphate binding cassette transporter A1
ApoA-I: Apolipoprotein A-I
CEC: Cholesterol efflux capacity
HDL: High-density lipoprotein
nHDL: Nascent high-density lipoprotein
LCAT: Lecithin-cholesterol acyltransferase
THP-1: Tamm-Horsfall protein 1
HAVICs: Human aortic valve interstitial cells
LDL: Low-density lipoprotein
GAPDH: Glyceraldehyde 3-phosphate dehydrogenase
PMA: Phorbol 12-myristate 13-acetate
2D-PAGGE: Two-dimensional non-denaturing gradient gel electrophoresis

## References

[1] Goody PR, Hosen MR, Christmann D, Niepmann ST, Zietzer A, Adam M, Bönner F, Zimmer S, Nickenig G, Jansen F (2020). Aortic valve stenosis: from basic mechanisms to novel therapeutic targets. Arterioscler Thromb Vasc Biol.

[2] Owens DS, Bartz TM, Buzkova P, Massera D, Biggs ML, Carlson SD, Psaty BM, Sotoodehnia N, Gottdiener JS, Kizer JR (2021). Cumulative burden of clinically significant aortic stenosis in community-dwelling older adults. Heart.

[3] Yi B, Zeng W, Lv L, Hua P (2021). Changing epidemiology of calcific aortic valve disease: 30-year trends of incidence, prevalence, and deaths across 204 countries and territories. Aging..

[4] Clavel MA, Pibarot P, Messika-Zeitoun D, Capoulade R, Malouf J, Aggarval S, Araoz PA, Michelena HI, Cueff C, Larose E, Miller JD, Vahanian A, Enriquez-Sarano M (2014). Impact of aortic valve calcification, as measured by mdct, on survival in patients with aortic stenosis: results of an international registry study. J Am Coll Cardiol.

[5] Henein M, Hällgren P, Holmgren A, Sörensen K, Ibrahimi P, Kofoed KF, Larsen LH, Hassager C (2015). Aortic root, not valve, calcification correlates with coronary artery calcification in patients with severe aortic stenosis: a two-center study. Atherosclerosis..

[6] Nasir K, Katz R, Al-Mallah M, Takasu J, Shavelle DM, Carr JJ, Kronmal R, Blumenthal RS, O'Brien K, Budoff MJ (2010). Relationship of aortic valve calcification with coronary artery calcium severity: the multi-ethnic study of atherosclerosis (mesa). J Cardiovasc Comput Tomogr.

[7] Lerman DA, Prasad S, Alotti N (2015). Calcific aortic valve disease: molecular mechanisms and therapeutic approaches. Eur Cardiol.

[8] Zhao Y, Nicoll R, He YH, Henein MY (2016). The effect of statins on valve function and calcification in aortic stenosis: a meta-analysis. Atherosclerosis..

[9] Smith JG, Luk K, Schulz CA, Engert JC, Do R, Hindy G, Rukh G, Dufresne L, Almgren P, Owens DS, Harris TB, Peloso GM, Kerr KF, Wong Q, Smith AV, Budoff MJ, Rotter JI, Cupples LA, Rich S, Kathiresan S, Orho-Melander M, Gudnason V, O'Donnell CJ, Post WS, Thanassoulis G (2014). Association of low-density lipoprotein cholesterol-related genetic variants with aortic valve calcium and incident aortic stenosis. JAMA.

[10] Stewart BF, Siscovick D, Lind BK, Gardin JM, Gottdiener JS, Smith VE, Kitzman DW, Otto CM (1997). Clinical factors associated with calcific aortic valve disease. Cardiovascular health study. J Am Coll Cardiol.

[11] Thanassoulis G, Campbell CY, Owens DS, Smith JG, Smith AV, Peloso GM, Kerr KF, Pechlivanis S, Budoff MJ, Harris TB, Malhotra R, O'Brien KD, Kamstrup PR, Nordestgaard BG, Tybjaerg-Hansen A, Allison MA, Aspelund T, Criqui MH, Heckbert SR, Hwang SJ, Liu Y, Sjogren M, van der Pals J, Kalsch H, Muhleisen TW, Nothen MM, Cupples LA, Caslake M, Di Angelantonio E, Danesh J, Rotter JI, Sigurdsson S, Wong Q, Erbel R, Kathiresan S, Melander O, Gudnason V, O'Donnell CJ, Post WS (2013). Genetic associations with valvular calcification and aortic stenosis. N Engl J Med.

[12] Summerhill VI, Moschetta D, Orekhov AN, Poggio P, Myasoedova VA. Sex-specific features of calcific aortic valve disease. Int J Mol Sci. 2020;21. 10.3390/ijms21165620. 10.3390/ijms21165620 PMC7460640 32781508

[13] Aggarwal SR, Clavel MA, Messika-Zeitoun D, Cueff C, Malouf J, Araoz PA, Mankad R, Michelena H, Vahanian A, Enriquez-Sarano M (2013). Sex differences in aortic valve calcification measured by multidetector computed tomography in aortic stenosis. Circ Cardiovasc Imaging.

[14] Linde L, Carter-Storch R, Christensen NL, Øvrehus KA, Diederichsen ACP, Laursen K, Jensen PS, Rasmussen LM, Møller JE, Dahl JS (2021). Sex differences in aortic valve calcification in severe aortic valve stenosis: association between computer tomography assessed calcification and valvular calcium concentrations. Eur Heart J Cardiovasc Imaging.

[15] Thaden JJ, Nkomo VT, Suri RM, Maleszewski JJ, Soderberg DJ, Clavel M-A, Pislaru SV, Malouf JF, Foley TA, Oh JK, Miller JD, Edwards WD, Enriquez-Sarano M (2016). Sex-related differences in calcific aortic stenosis: correlating clinical and echocardiographic characteristics and computed tomography aortic valve calcium score to excised aortic valve weight. Eur Heart J.

[16] Parra-Izquierdo I, Castaños-Mollor I, López J, Gómez C, San Román JA, Sánchez Crespo M, García-Rodríguez C (2018). Calcification induced by type i interferon in human aortic valve interstitial cells is larger in males and blunted by a janus kinase inhibitor. Arterioscler Thromb Vasc Biol.

[17] Parra-Izquierdo I, Castaños-Mollor I, López J, Gómez C, San Román JA, Sánchez Crespo M, García-Rodríguez C (1865). Lipopolysaccharide and interferon-γ team up to activate hif-1α via stat1 in normoxia and exhibit sex differences in human aortic valve interstitial cells. Biochim Biophys Acta Mol basis Dis.

[18] Annabi MS, Clisson M, Fleury MA, Voisine M, Hervault M, Shen M, Boilard AJ, Marette A, Ong G, Côté N, Clavel MA (2020). Sex-differences in echocardiographic assessment of aortic valve in young adult ldlr(−/−)/apob(100/100)/igf-ii(+/−) mice. Exp Gerontol.

[19] Arsenault BJ, Dube MP, Brodeur MR, de Oliveira Moraes AB, Lavoie V, Kernaleguen AE, Guauque-Olarte S, Mathieu P, Pibarot P, Messika-Zeitoun D, Bosse Y, Rhainds D, Rheaume E, Tardif JC (2014). Evaluation of links between high-density lipoprotein genetics, functionality, and aortic valve stenosis risk in humans. Arterioscler Thromb Vasc Biol.

[20] Khera AV, Demler OV, Adelman SJ, Collins HL, Glynn RJ, Ridker PM, Rader DJ, Mora S (2017). Cholesterol efflux capacity, high-density lipoprotein particle number, and incident cardiovascular events: an analysis from the Jupiter trial (justification for the use of statins in prevention: an intervention trial evaluating rosuvastatin). Circulation..

[21] Khera AV, Cuchel M, de la Llera-Moya M, Rodrigues A, Burke MF, Jafri K, French BC, Phillips JA, Mucksavage ML, Wilensky RL, Mohler ER, Rothblat GH, Rader DJ (2011). Cholesterol efflux capacity, high-density lipoprotein function, and atherosclerosis. N Engl J Med.

[22] Rohatgi A, Khera A, Berry JD, Givens EG, Ayers CR, Wedin KE, Neeland IJ, Yuhanna IS, Rader DR, de Lemos JA, Shaul PW (2014). Hdl cholesterol efflux capacity and incident cardiovascular events. N Engl J Med.

[23] Saleheen D, Scott R, Javad S, Zhao W, Rodrigues A, Picataggi A, Lukmanova D, Mucksavage ML, Luben R, Billheimer J, Kastelein JJ, Boekholdt SM, Khaw KT, Wareham N, Rader DJ (2015). Association of hdl cholesterol efflux capacity with incident coronary heart disease events: a prospective case-control study. Lancet Diabetes Endocrinol.

[24] Vaughan AM, Oram JF (2003). Abca1 redistributes membrane cholesterol independent of apolipoprotein interactions. J Lipid Res.

[25] Busseuil D, Shi Y, Mecteau M, Brand G, Kernaleguen AE, Thorin E, Latour JG, Rheaume E, Tardif JC (2008). Regression of aortic valve stenosis by apoa-i mimetic peptide infusions in rabbits. Br J Pharmacol.

[26] Lommi JI, Kovanen PT, Jauhiainen M, Lee-Rueckert M, Kupari M, Helske S (2011). High-density lipoproteins (hdl) are present in stenotic aortic valves and may interfere with the mechanisms of valvular calcification. Atherosclerosis..

[27] Schlotter F, Halu A, Goto S, Blaser MC, Body SC, Lee LH, Higashi H, DeLaughter DM, Hutcheson JD, Vyas P, Pham T, Rogers MA, Sharma A, Seidman CE, Loscalzo J, Seidman JG, Aikawa M, Singh SA, Aikawa E (2018). Spatiotemporal multi-omics mapping generates a molecular atlas of the aortic valve and reveals networks driving disease. Circulation..

[28] Lim J, Aguilan JT, Sellers RS, Nagajyothi F, Weiss LM, Angeletti RH, Bortnick AE (2020). Lipid mass spectrometry imaging and proteomic analysis of severe aortic stenosis. J Mol Histol.

[29] Goto S, Rogers MA, Blaser MC, Higashi H, Lee LH, Schlotter F, Body SC, Aikawa M, Singh SA, Aikawa E (2019). Standardization of human calcific aortic valve disease in vitro modeling reveals passage-dependent calcification. Front Cardiovasc Med.

[30] Kocyigit D, Zimetti F, Gurses KM, Zanotti I, Marchi C, Ståhlman M, Borén J, Canpinar H, Soyal MFT, Guc D, Hazirolan T, Ozer N, Tokgozoglu L (2021). Cholesterol efflux promoting function of high-density lipoproteins in calcific aortic valve stenosis. Atherosclerosis Plus.

[31] Kritharides L, Christian A, Stoudt G, Morel D, Rothblat GH (1998). Cholesterol metabolism and efflux in human thp-1 macrophages. Arterioscler Thromb Vasc Biol.

[32] Mulya A, Lee JY, Gebre AK, Thomas MJ, Colvin PL, Parks JS (2007). Minimal lipidation of pre-beta hdl by abca1 results in reduced ability to interact with abca1. Arterioscler Thromb Vasc Biol.

[33] Chen CH, Albers JJ. Characterization of proteoliposomes containing apoprotein A-I: a new substrate for the measurement of lecithin: cholesterol acyltransferase activity. J Lipid Res. 1982;23(5):680–91. 6811681

[34] Miida T, Miyazaki O, Hanyu O, Nakamura Y, Hirayama S, Narita I, Gejyo F, Ei I, Tasaki K, Kohda Y, Ohta T, Yata S, Fukamachi I, Okada M (2003). Lcat-dependent conversion of prebeta1-hdl into alpha-migrating hdl is severely delayed in hemodialysis patients. J Am Soc Nephrol.

[35] Dobiásová M, Frohlich J (1996). Measurement of fractional esterification rate of cholesterol in plasma depleted of apoprotein b containing lipoprotein: methods and normal values. Physiol Res.

[36] Boyer M, Mitchell PL, Poirier P, Alméras N, Tremblay A, Bergeron J, Després J-P, Arsenault BJ (2018). Impact of a one-year lifestyle modification program on cholesterol efflux capacities in men with abdominal obesity and dyslipidemia. Am J Physiol Endocrinol Metab.

[37] Au - Lin C, Au - Zhu D, Au - Markby G, Au - Corcoran BM, Au - Farquharson C, Au - Macrae VE. Isolation and characterization of primary rat valve interstitial cells: a new model to study aortic valve calcification. JoVE. 2017:e56126. 10.3791/56126. 10.3791/56126 PMC5755457 29286439

[38] Duong PT, Weibel GL, Lund-Katz S, Rothblat GH, Phillips MC (2008). Characterization and properties of pre beta-hdl particles formed by abca1-mediated cellular lipid efflux to apoa-i. J Lipid Res.

[39] Akers EJ, Nicholls SJ, Di Bartolo BA (2019). Plaque calcification: Do lipoproteins have a role?. Arterioscler Thromb Vasc Biol.

[40] Wang H, Goland S, Burton K, Czer LS, Schwarz ER, Trento A (2010). An agonist of liver x receptor slows valvular disease in a hypercholesterolemia mouse model. J Heart Valve Dis.

[41] Sutherland WHF, Temple WA, Nye ER (1979). Lecithin:Cholesterol acyltransferase activity, plasma and lipoprotein lipids and obesity in men and women. Atherosclerosis..

[42] Rousset X, Vaisman B, Amar M, Sethi AA, Remaley AT (2009). Lecithin: Cholesterol acyltransferase--from biochemistry to role in cardiovascular disease. Curr Opin Endocrinol Diabetes Obesity.

[43] Sacks FM, Liang L, Furtado JD, Cai T, Davidson WS, He Z, McClelland RL, Rimm EB, Jensen MK (2020). Protein-defined subspecies of hdls (high-density lipoproteins) and differential risk of coronary heart disease in 4 prospective studies. Arterioscler Thromb Vasc Biol.

[44] Davidson WS, Cooke AL, Swertfeger DK, Shah AS (2021). The difference between high-density lipoprotein subfractions and subspecies: an evolving model in cardiovascular disease and diabetes. Curr Atheroscler Rep.

[45] Sritharen Y, Enriquez-Sarano M, Schaff HV, Casaclang-Verzosa G, Miller JD (2017). Pathophysiology of aortic valve stenosis: is it both fibrocalcific and sex specific?. Physiology (Bethesda, Md).

[46] Rubinow KB, Vaisar T, Chao JH, Heinecke JW, Page ST (2018). Sex steroids mediate discrete effects on hdl cholesterol efflux capacity and particle concentration in healthy men. J Clin Lipidol.

[47] Badeau RM, Metso J, Kovanen PT, Lee-Rueckert M, Tikkanen MJ, Jauhiainen M (2013). The impact of gender and serum estradiol levels on hdl-mediated reverse cholesterol transport. Eur J Clin Investig.

[48] Masjedi S, Lei Y, Patel J, Ferdous Z (2017). Sex-related differences in matrix remodeling and early osteogenic markers in aortic valvular interstitial cells. Heart Vessel.

