# Supplementary material for: Measures of high-density lipoprotein function in men and women with severe aortic stenosis
Source: Lipids Health Dis. 2022 May 28;21:48. doi: 10.1186/s12944-022-01653-7 (PMC9148512; doi:10.1186/s12944-022-01653-7)
Supplement: Supplementary file 1 — Additional file 1. [file 12944_2022_1653_MOESM1_ESM.docx]

**Supplementary Information**

Additional file 1 of step to validation of cholesterol efflux from cell cultures THP-1 and HAVIC to control plasma HDL.

**
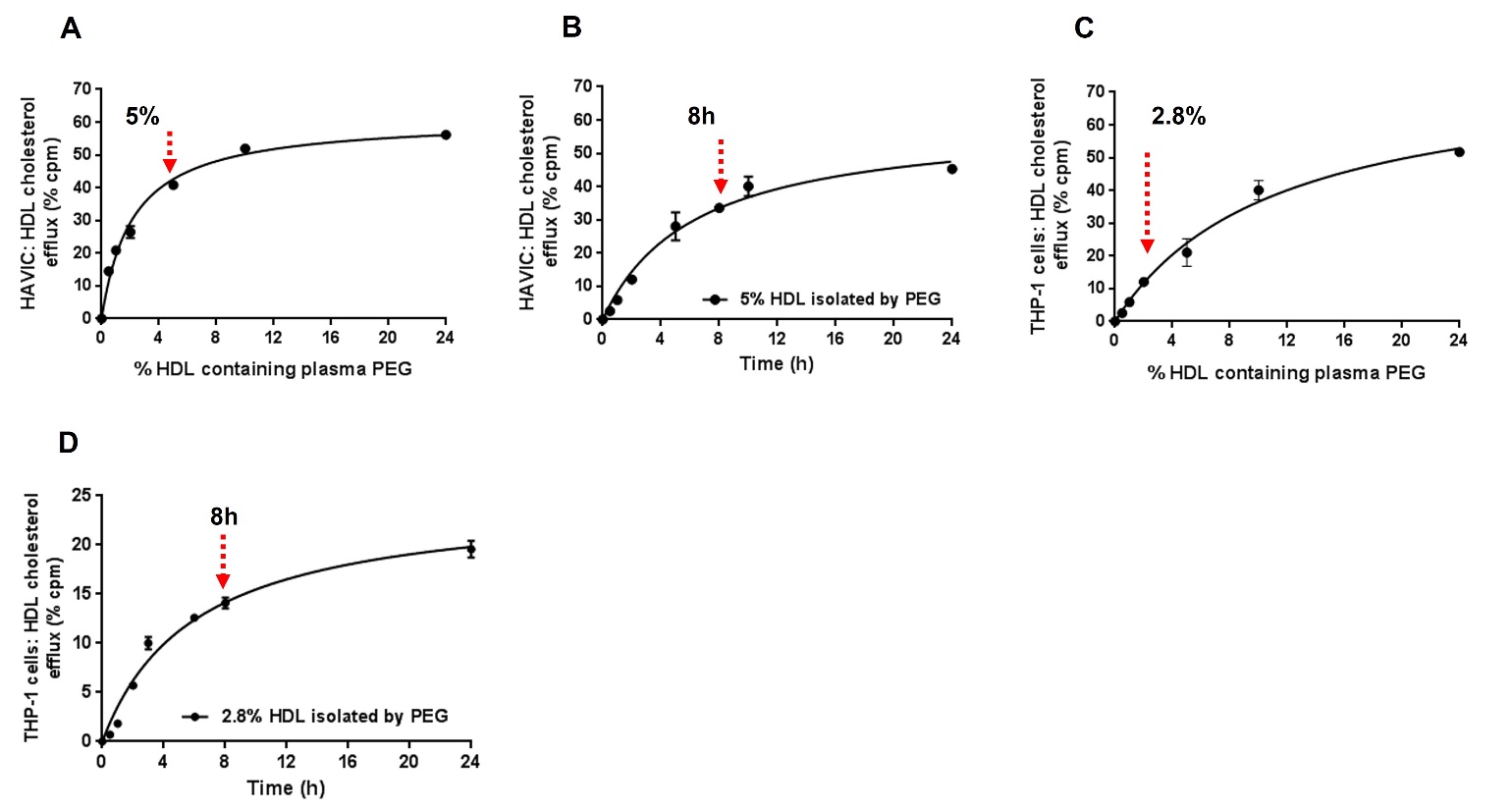
**

**Figure 1**. Characteristics of cholesterol efflux from human aortic vascular interstitial cells (HAVICs) and Tamm-Horsfall protein 1 (THP-1) macrophages to control plasma high-density lipoprotein (HDL). (**A**) Dose-course of ABCA1-mediated cholesterol efflux to control HDL isolated from pooled human plasma from non-calcified HAVICS. Red arrow indicates optimum dose (5%). (**B**) Time-course activity of ABCA1-mediated cholesterol efflux to control HDL (5%) incubated with non-calcified HAVICS. Cholesterol efflux was determined at the time points indicated by the red arrow. **(C)** Cholesterol efflux to increased doses of control HDL in THP-1 macrophages. Red arrow indicates optimum dose (2.8%). **(D)** Time course cholesterol efflux in THP-1 macrophages to control HDL (2.8%).

**Table 1.** Kinetics for cholesterol efflux to control plasma HDL from Tamm-Horsfall protein 1 (THP-1) macrophages and human aortic valve interstitial cells (HAVICs).

| **THP-1** | | **non-calcified HAVICs** | | **Calcified HAVICs** | |
| --- | --- | --- | --- | --- | --- |
| *V_max_* | *K_m_* | *V_max_* | *K_m_* | *V_max_* | *K_m_* |
| 38.67±1.81 | 1.66±0.19% | 63.03±2.50 | 0.64±0.24% | 30.06±2.36 | 2.30±0.30% |

HAVICs, human aortic valve interstitial cells; HDL, high-density lipoprotein; *K_m_*, efflux efficiency, % HDL/mL; THP-1, Tamm-Horsfall protein 1 macrophages; *V_max,_* maximum velocity, % efflux/24h for ABCA1-mediated efflux.
